# A Review of Intense Pulsed Light in the Treatment of Ocular Rosacea

**DOI:** 10.1177/12034754241254051

**Published:** 2024-05-28

**Authors:** Mahek Shergill, Sophie Khaslavsky, Shani Avraham, Nadia Kashetsky, Kirill Zaslavsky, Ilya Mukovozov

**Affiliations:** 1 Michael G. DeGroote School of Medicine, McMaster University, Hamilton, ON, Canada; 2 Vancouver General Hospital, Vancouver, BC, Canada; 3 Foresee Eyecare, Toronto, ON, Canada; 4 Department of Dermatology and Skin Science, University of British Columbia, Vancouver, BC, Canada; 5 Department of Ophthalmology and Vision Sciences, University of Toronto, Toronto, ON, Canada; 6 Toronto Dermatology Centre, Toronto, ON, Canada

**Keywords:** rosacea, ocular, intense pulsed light, treatment

## Abstract

**Introduction:**

Ocular rosacea is an underdiagnosed form of rosacea that may occur with or without typical cutaneous signs of rosacea. One of the common manifestations is dry eyes. Although the use of intense pulsed light (IPL) in the treatment of rosacea-related dry eyes has been reported, a recent review is lacking.

**Methods:**

A scoping review was performed to summarize the efficacy of IPL in the treatment of ocular rosacea.

**Results:**

Five articles were included, representing 108 patients, with a mean age of 58.4 years. Based on available data, 59.2% (n = 58/98) were female. The studies detailed the use of IPL in combination with meibomian gland expression treatment. Overall, 91% (n = 89/98) of patients with ocular rosacea treated with IPL had a partial response and 9% (n = 9/98) had no response. IPL therapy did not lead to complete recovery in any of the included patients. One participant experienced an adverse event across the included studies.

**Conclusions:**

IPL is a promising treatment modality for ocular rosacea, as demonstrated by its ability to relieve dry eye symptoms with limited adverse events. Further research into this novel treatment is necessary to ascertain its role in the management of ocular rosacea.

## Introduction

Rosacea is a chronic, inflammatory skin condition characterized by flushing, erythema, papules, pustules, and telangiectasia.^1^ The prevalence of rosacea has been estimated to be about 10% of the population, with a higher incidence in those with fair skin.^2^ It is 3 times more common in women than men.^2^ The pathogenic mechanism of rosacea is multifaceted, with factors such as genetics, as well as exposure to environmental triggers such as ultraviolet light, temperature changes, spicy foods, and stress all linked to its development.^3^

Ocular symptoms are present in an estimated 58% to 72% of rosacea patients.^4^ Ocular rosacea may occur with or without facial symptoms of rosacea, making it difficult to diagnose.^2,5^ Common clinical findings include conjunctival hyperemia, meibomian gland dysfunction, and corneal vascularization.^4^ Meibomian gland dysfunction manifests in the form of itching and dry eye sensations. Mild ocular rosacea primarily involves the eyelid margin and meibomian gland, moderate disease involves the ocular surface, and severe disease includes corneal involvement.^4^ When severe cases are left untreated, ocular rosacea may lead to poor irreversible outcomes, such as vision loss.^4^

Current guidelines for the treatment of ocular rosacea outline the use of 50 to 100 mg of doxycycline twice daily.^6^ The use of light-based therapy in the treatment of ocular rosacea is an emerging avenue and could potentially be a safe and noninvasive mode of therapy. In addition, the use of light-based treatment avoids microbiome alterations associated with antibiotic use, contributing to the promotion of antimicrobial stewardship. Intense pulsed light (IPL) therapy devices use flashlamps to produce pulsed light of different wavelengths, durations, and fluences.^7^

The treatment protocol demonstrating the use of IPL for dry eyes was first outlined by Dr Ronaldo Toyos.^8^ The process begins with the use of IPL eye pads over closed eyes.^8^ Then ultrasound gel is applied on the patient’s face from tragus to tragus, and overlapping flashes are delivered over the area to encompass one full pass.^8^ Subsequently, more ultrasound gel is applied, and a second pass is performed.^8^ Next, the gel is removed and 1% proparacaine is administered prior to beginning gland expression.^8^ Using a sterile cotton tip, it is placed on the area of the palpebral conjunctiva, and the physician places a finger over the skin adjacent to this gland.^8^ With the patient looking up, a gentle consistent pressure is applied with both the cotton tip and finger, and the gland is expressed for 30 seconds.^8^ This process is repeated across the span of the lower lid bilaterally.^8^ Gland expression of the upper eyelid is performed using finger pressure while the patient is instructed to look down.^8^ If finger pressure does not suffice, a sterile cotton tip can also be used.^8^ On completion of gland expression, a drop of topical steroid or a nonsteroidal anti-inflammatory drug can be administered.^8^ The protocol encompasses 4 IPL treatments followed by meibomian gland expression (MGX) 4 to 6 weeks apart.^8^

Although the exact mechanism of action of IPL in ocular rosacea is unknown, it is proposed to employ the benefits of heat-inducing ablation.^9^ One proposed mechanism highlights the warming effect produced by IPL as integral to increasing the expression and subsequent outflow of viscous meibum found in conditions causing dysfunctional meibum glands.^9^ The increased outflow of meibum reduces the proliferation of bacteria typically seen in meibum stasis, therefore decreasing ocular inflammation and irritation.^9^ Another potential explanation is the targeting and subsequent reduction of *Demodex* mites which have been found to contribute to inflammation.^9^ Finally, IPL may have a role in altering the levels of inflammatory mediators, specifically through the downregulation of pro-inflammatory cytokines and upregulation of anti-inflammatory cytokines.^9^ IPL may also induce changes in the microcirculation, through the eradication of abnormal small vessels surrounding meibomian glands, ultimately decreasing the availability of inflammatory mediators and reducing inflammation.^9^

IPL is a promising avenue of treatment and warrants further investigation to determine its impact on ocular rosacea outcomes.

## Methods

A scoping review of the literature was conducted.

### Search Strategy

A literature search was conducted on January 12, 2022, using the MEDLINE (1946 to January 11, 2022), Embase (1980 to January 11, 2022), and PubMed databases using the search terms “rosacea” and (“treatment” or “therapy” or “management” or “IPL” or “light”). No date, geographical, or language restrictions were applied.

### Study Eligibility Criteria

Eligibility criteria for this review were established using the population, intervention, comparator, outcomes, and study design (PICOS) framework. Studies were included if they met the following predetermined criteria:

- *Population*: individuals aged ≥18 years old diagnosed with ocular rosacea by a healthcare professional, including individuals with any disease severity, treatment history, or comorbid conditions
- *Intervention*: any treatment regimen involving light therapy
- *Comparator*: untreated individuals with ocular rosacea or in the absence of a comparator, open-label studies where outcomes were assessed in treated individuals
- *Outcomes*: clinical response (patient or physician reported) on dry eye symptoms, length of delay to diagnosis, timing of ocular, and cutaneous symptom presentation
- *Study design*: randomized control trials, cohort studies, cross-sectional studies, and case series (excluded if <3 subjects).

### Data Screening

Title and abstract screening were completed by 3 independent researchers (S.K., S.A., and I.M.) using Covidence online systematic review software (www.covidence.org). Conflicts were resolved by consensus and ties were broken by a third researcher (I.M.). Full-text review was completed independently by 3 researchers (S.K., S.A., and I.M.) and conflicts were resolved by consensus. References of included studies were manually checked to identify any additional relevant studies.

### Data Extraction

Data extraction was completed in duplicate by 3 reviewers (S.K., S.A., and N.K.) using a predetermined extraction form including study characteristics (publication year, country, sample size), patient characteristics (age, biological sex of participants), intervention type, and outcomes (clinical response and length of follow-up).

### Data Synthesis

To aid in pooling results from heterogenic outcomes, outcomes from each study were coded into 3 categories: complete response (CR), partial response (PR), and no response (NR).

### Definitions

*Complete response*: complete or near complete response based on criteria established by each included study.

*Partial response*: partial or moderate response based on criteria established by each study.

*No response*: no response or worsening symptoms based on criteria established by each study.

## Results

Five articles met the inclusion criteria and are summarized in this review (Supplemental Table S1). The mean age of participants was 58.4 years. All the included studies were conducted in adult populations, and the follow-up period ranged from 10 weeks to 12 months.

Overall IPL therapy led to a PR in 91% (n = 89/98) of included patients, and 9% (n = 9/98) had NR. IPL therapy did not lead to complete recovery in any of the included patients. The Sagaser et al study was included in our review but was excluded from the pooled analysis as it did not provide information on response per participant.

### Summary of Included Studies

All included studies reported an improvement in ocular rosacea symptoms. A study investigating the use of IPL and MGX in chronic graft versus host disease (GVHD) patients with ocular rosacea noted a decrease in the Standard Patient Evaluation of Eye Dryness Questionnaire (SPEED2) score measuring the severity of dry eye disease.^10^ This study used the treatment protocol first developed by Dr Rolando Toyos, which outlines a total of 4 IPL treatments followed by MGX 4 to 6 weeks apart.^8^ Among 8 patients, where the baseline score was 20.7, a statistically significant mean improvement of 8.0 points in the SPEED2 score was noted and this improvement was maintained after 12 months.^10^ However, meibomian gland evaluation did not reveal any change in the number of meibomian glands producing liquid secretions over the 12 month period.^10^ One adverse event was reported, where the patient withdrew from the study due to the development of active GVHD.^10^ Similarly, in another study where participants received an average of 3 IPL sessions, SPEED2 measurements found that 62.3% of participants experienced a greater than 50% reduction in SPEED2, and overall, 85% of patients reported a positive SPEED2 response to IPL therapy.^11^ In contrast to the previous study, this study observed an increase in MGX, where 71% of patients had improvement in the number of glands with liquid secretions in the lower eyelid.^11^

In a group of 20 patients with dry eye disease associated with ocular rosacea, a statistically significant improvement in subjective symptom score was noted, from a median value of 8 to 2.^12^ Tear breakup time, a metric of tear film stability, changed from a median of 4 to 10 following therapy.^12^ Other statistically significant changes included the Schirmer test result from 13.5 to 15, and the Van Bijsterveld score remained at 3.^12^ The treatment algorithm comprised of 3 sessions including warm eyelid compresses for 7 minutes, manual MGX, administration of 5 flashes of xenon light, and ending with a second manual MGX.^12^

In a study investigating the use of IPL in rosacea-associated meibomian gland dysfunction, the Ocular Surface Disease Index (OSDI) score was used to characterize treatment outcomes.^13^ A total of 82.4% of the participants reported an improvement in symptoms between the baseline and final examination, and the remaining participants described improvement within the follow-up period.^13^ The OSDI is a validated and reliable tool used to measure the severity of dry eye disease.^14^ It is scored on a scale from 0 to 100, where higher scores correspond to greater severity of disease—a score of 0 to 12 is characterized as normal, 13 to 15 is mild disease, 23 to 32 as moderate, and 33 to 100 as severe.^14^ The baseline OSDI score was noted to be 50.0, and at 12 months was <40.0.^13^ Other outcome metrics also described improvement, where tear film instability and ocular surface epithelial damage resolved during treatment and 3 weeks posttreatment.^13^ A study by Sagaser et al. also found that while the mean OSDI scores between the IPL-MGX and MGX treatment groups were comparable at baseline (55.7 vs 43.5, *P* = .212), following 4 visits, the OSDI scores in the IPL-MGX group were significantly improved compared to the MGX only group (*P* = .030).^15^ Similar to previously described studies, these studies used the Toyos treatment protocol.

## Discussion

Our review summarizes studies reporting on the use of IPL for the treatment of ocular rosacea. Available data suggest that IPL can lead to an improvement of symptoms associated with ocular rosacea, particularly dry eyes, with limited adverse events, as evidenced by the PR to treatment demonstrated in 91% (n = 89/98) of included patients. Given the chronic nature of rosacea and lack of curative treatment, the results similarly demonstrate no CR. PR was variable, but primarily encompassed decreased frequency and severity of dry eye symptoms such as dryness, irritation, burning, watering, and eye fatigue, as well as reduced redness of the eyelid margins. Interestingly, this treatment effect is demonstrated despite no direct application of IPL to the eyelids. Furthermore, though IPL application is limited up to the margins of the lower eyelid shields, there is improvement in both the lower and upper eyelids. This could be due to the increase in anti-inflammatory mediators and antioxidants that are being propagated through the orbital vasculature.^16^ There are updated protocols treating the upper eyelid as well, specifically using a Jaeger lid plate beneath the conjunctival sac as the IPL is pulsed, or using an internal eye shield as a means to protect the cornea and conjunctival sac and to mitigate adverse effects secondary to the IPL.^17,18^ There are also published studies on the use of external adhesive eye shields that can be placed to protect the upper and lower lashes, exposing the upper lid and using a handpiece with a smaller surface area to directly target the upper meibomian glands.^17^

The literature demonstrates that IPL applied to the eyelids in addition to MGX is a relatively safe procedure with low risk of adverse effects. Some adverse events reported are epidermal keratoconjunctivitis, floaters, herpes simplex keratitis, glaucomatocyclitic crisis, and iridocyclitis. IPL is not recommended for children <10 years of age or patients who have underlying uveitis or glaucomatocyclitic crisis. Caution is advised in those with a history of herpes simplex keratitis.^19^ There are no studies to compare efficacy between treating lower eyelids and treating both upper and lower eyelids. Caution should be used when treating the upper eyelid as partial eyelash loss has been reported.^17^ Conflicting evidence exists regarding the potential increase in MGX with IPL; therefore, more research is warranted to determine whether IPL can lead to consistent physical changes and symptom improvement.

A correlation between ocular rosacea and the presence of bacteria associated with *Demodex* mites has been described in the literature.^15^ *Demodex* mites are parasites that normally colonize human hair follicles and sebaceous glands, but a greater density of these mites has been associated with the pathophysiology of rosacea and other inflammatory skin conditions.^20^ A study of 59 participants by Li et al^20^ found that positive serum immunoreactivity to *Bacillus oleronius* was significantly associated with lid margin inflammation (*P* = .040) and ocular *Demodex* infestation (*P* = .048). *Demodex* infestation can lead to ocular surface inflammation, causing blepharitis, meibomian gland dysfunction, and conjunctivitis.^20^ Given the pathogenic role of *Demodex*, it is plausible that IPL targets the eradication of mites for symptomatic relief.^9^

*Demodex* mites and their associated bacteria have also been linked to meibomian gland pathology.^21^ These organisms enhance the production of lipases and esterases degrading the lipid content of meibum, a key component of the tear film.^22^ This causes the meibum to become more viscous, causing stasis of the meibum which subsequently leads to lowered secretion.^22^ The compromised tear film lipid layer causes tear film instability and dry eyes.^22^ Thus, IPL could heat the meibum, improving stasis and increasing MGX.^9^

Though the number of sessions and time between sessions varied across studies, 3 to 4 treatments were most commonly administered and spaced 4 to 6 weeks apart. The IPL application methods used are summarized in this review (Supplemental Table S2). Many of the included studies described the Toyos protocol, encompassing 4 IPL treatments followed by MGX 4 to 6 weeks apart.^8^ This protocol describes that candidates for IPL must have Fitzpatrick skin types 1, 2, or 3, where sometimes type 4 can tolerate treatment.^8^ Clinicians should exercise caution in administering IPL in patients with Fitzpatrick skin types 4 to 7. IPL treatment intensity ranges from 8 to 20 J/cm^2^, where higher intensities may be employed based on increasing age and lid margin disease severity.^8^ The number of sessions required for maintenance was not reported in the included studies and more research is needed before the development of recommendations.

There are limitations to this review. Our study categorized participant outcomes as producing a CR, PR, or NR as an aggregate measure allowing for the pooling of heterogeneous modalities. A limitation of this method is the variability of PRs, due to the spectrum of strong to weak PRs being grouped together. There is also variability in the patients of included studies and their comorbidities. Given the relatively novel nature of IPL as a form of therapy for ocular rosacea, there are few studies investigating its efficacy, and these studies often use small groups of participants.

## Conclusion

Our review highlights the potential for IPL in the management of dry eyes associated with ocular rosacea. Although a framework for treatment has been developed, further research is necessary to ascertain a treatment algorithm outlining the number of IPL sessions necessary depending on factors such as Fitzpatrick skin type, symptom severity, and age. Overall, IPL remains a potential treatment modality for symptom management in combination with appropriate lid hygiene and massage.

## Supplemental Material

sj-docx-1-cms-10.1177_12034754241254051 – Supplemental material for A Review of Intense Pulsed Light in the Treatment of Ocular Rosacea Supplemental material, sj-docx-1-cms-10.1177_12034754241254051 for A Review of Intense Pulsed Light in the Treatment of Ocular Rosacea by Mahek Shergill, Sophie Khaslavsky, Shani Avraham, Nadia Kashetsky, Kirill Zaslavsky and Ilya Mukovozov in Journal of Cutaneous Medicine and Surgery
