# Supplementary material for: A Review of Intense Pulsed Light in the Treatment of Ocular Rosacea
Source: J Cutan Med Surg. 2024 May 28;28(4):370–4. doi: 10.1177/12034754241254051 (PMC11403922; doi:10.1177/12034754241254051)
Supplement: sj-docx-1-cms-10.1177_12034754241254051 – Supplemental material for A Review of Intense Pulsed Light in the Treatment of Ocular Rosacea [file sj-docx-1-cms-10.1177_12034754241254051.docx]

**Table S1:** Summary table of included studies.

| Author, year | Treatment | Number of Patients | Sex (M:F) | Complete Response, n | Partial Response, n (%) | No Response, n (%) | Adverse Events, n | Follow Up |
| --- | --- | --- | --- | --- | --- | --- | --- | --- |
| *Mejia et al.*  *(2019)* | IPL + MGX: 3 sessions | 20 | N/A | 0 | 20 | 0 | None | 10 weeks |
| *Sagaser et al. (2021)* | IPL + MGX: 4 sessions | 10* | NR | - | - | - | None | NR |
| *Seo et al.*  *(2018)* | IPL + MGX: 4 sessions | 17 | 10:7 | 0 | 17 | 0 | None | 3 months |
| *Shen et al.*  *(2015)* | IPL + MGX: 4 sessions | 9** | 5:4 | 0 | 8 | 1 | Developed active GVHD, 1 | 12 months |
| *Vegunta et al.*  *(2018)* | IPL + MGX: 3 sessions (average) | 52 | 8:44 | 0 | 44 (85) | 8 (15) | None | NR |
| Summary |  | 108 | 40:58 | 0 | 89 (91) | 9 (9) | 1 | 11.3 weeks |

*This study did not report participant-specific response outcomes.

**1 patient reported an adverse event and withdrew from the study and was classified as a non-responder.

GVHD: graft versus host disease

**Table S2:** Summary table of IPL application method used by each study.

| *Author, year* | IPL Application Method |
| --- | --- |
| *Mejia et al. (2019)* | Warm eyelid compress for 7 minutes, followed by manual expression of meibomian glands. Then, conducting gel applied and 5 flashes of xenon light over the zygomatic area applied. Following this, manual gland expression was performed for the second time. |
| *Sagaser et al. (2021)* | The treatment protocol demonstrating the use of IPL for dry eyes was first outlined by Dr. Ronaldo Toyos.^8^ The process begins with the use of IPL eye pads over closed eyes.^8^ Then ultrasound gel is applied on the patient’s face from tragus to tragus, and overlapping flashes are delivered over the area to encompass one full pass.^8^ Subsequently, more ultrasound gel is applied, and a second pass is performed.^8^ Next, the gel is removed and 1% proparacaine is administered prior to beginning gland expression.^8^ Using a sterile cotton tip, it is placed on the area of the palpebral conjunctiva, and the physician places a finger over the skin adjacent to this gland.^8^ With the patient looking up, a gentle consistent pressure is applied with both the cotton tip and finger, and the gland is expressed for 30 seconds.^8^ This process is repeated across the span of the lower lid bilaterally.^8^ Gland expression of the upper eyelid is performed using finger pressure while the patient is instructed to look down.^8^ If finger pressure does not suffice, a sterile cotton tip can also be used.^8^ Upon completion of gland expression, a drop of topical steroid or a nonsteroidal anti-inflammatory drug can be administered.^8^ The protocol encompasses four IPL treatments followed by MGX 4-6 weeks apart.^8^ |
| *Seo et al. (2018)* | Technique described by Toyos et al. ^8^ |
| *Shen et al. (2015)* | Technique described by Toyos et al. ^8^ |
| Vegunta et al. (2018) | Technique described by Toyos et al. ^8^ |
